# 

**Published:** 1881-03

**Authors:** 


### EDITORIAL.

### MICHIGAN DENTAL SOCIETY.

The annual meeting of this body will be held in Detroit on Thursday and Friday, the 24th and 25th of this month (March).

The executive committee has made such arrangements as will make certain a good meeting, if the members of the Society and profession will do their part by being present and by taking up the line of work laid out.

This is one of the oldest State Societies in the country, and its record is one of which the profession in Michigan should be proud.

The following programme prepared, viz. :

1st. Oral Surgery.

2nd. Operative Dentistry.

3rd. Voluntary Essays.

4th. Mechanical Dentistry.

5th. Clinics in Operative Dentistry.

6th. Dentistry, its relation to Hygiene and the Food.

It is desirable that preparation upon each of these subjects should be made by the members, in order that the greatest good may be derived from the meeting.

A MONTHLY MAGAZINE DEVOTED TO THE INTERESTS OF

**THE DENTAL PROFESSION.**

*TERMS REDUCED TO $2.50 PER YEAR. SINGLE COPIES 25C.*

EDITED BY PROF. J. TAFT, D.D.S.

AND

*Published by SPENCER, CROCKER & CO., Ohio Dental Depot.*

The volume commences in January and closes in December.

The REGISTER being issued monthly is always fresh, therefore Indispensable to the practitioner who desires to keep posted up with the new inventions and improvements which are daily developed. Neither expense nor effort will be spared to give value and variety to its contents. Articles will be illustrated with well executed engravings whenever they will add to the interest or assist to explanation.

Its extensive circulation and frequency of issue make it one of the BEST DENTAL ADVERTISING MEDIUMS in the United States.

All subscriptions must begin with January or July.

Local or special notices, five lines or less, will be inserted for$3 each insertion ; over five lines, 50 cts. per line.

All advertising bills payable in advance, or at furthest, after the issue of the first number containing the advertisement. All transient advertisements to be paid for in advance.

☞CONTRIBUTIONS SOLICITED.

Exclusively Editorial Communications should be addressed to Editor.

The latest number of the REGISTER may be ordered with or without oth-3 goods at any time, and all letters should be addressed to

*SPENCER, CROCKER & CO., Ohio Dental Depot, Cincinnati, O.*

